# Genetic variation drives cancer cell adaptation to ECM stiffness

**DOI:** 10.1073/pnas.2403062121

**Published:** 2024-09-20

**Authors:** Ting-Ching Wang, Suchitaa Sawhney, Daylin Morgan, Richard L. Bennett, Richa Rashmi, Marcos R. Estecio, Amy Brock, Irtisha Singh, Charles F. Baer, Jonathan D. Licht, Tanmay P. Lele

**Affiliations:** ^a^Artie McFerrin Department of Chemical Engineering, Texas A&M University, College Station, TX 77843; ^b^Department of Biomedical Engineering, Texas A&M University, College Station, TX 77843; ^c^Department of Biomedical Engineering, The University of Texas at Austin, Austin, TX 78712; ^d^Division of Hematology and Oncology, University of Florida Health Cancer Center, Gainesville, FL 32610; ^e^Department of Cell Biology and Genetics, Texas A&M University, Bryan, TX 77807; ^f^Department of Epigenetics and Molecular Carcinogenesis, The University of Texas MD Anderson Cancer Center, Houston, TX 77030; ^g^Department of Biology, University of Florida, Gainesville, FL 32611; ^h^Department of Translational Medical Sciences, Texas A&M University, Houston, TX 77030

**Keywords:** cancer, mechanobiology, ECM stiffness, mechanoadaptation

## Abstract

The progression of many solid tumors is accompanied by temporal and spatial changes in the stiffness of the extracellular matrix (ECM). Cancer cells adapt to soft and stiff ECM through mechanisms that are not fully understood. It is well known that there is significant genetic heterogeneity from cell to cell in tumors, but how ECM stiffness as a parameter might interact with that genetic variation is not known. Here, we employed experimental evolution to study the response of genetically variable and clonal populations of tumor cells to variable ECM stiffness. Proliferation rates of genetically variable populations cultured on soft ECM increased over a period of several weeks, whereas clonal populations did not evolve. Tracking of DNA barcoded cell lineages revealed that soft ECM consistently selected for the same few variants. These data provide evidence that ECM stiffness exerts natural selection on genetically variable tumor populations. Soft-selected cells were highly migratory, with enriched oncogenic signatures and unusual behaviors such as spreading and traction force generation on ECMs with stiffness as low as 1 kPa. Rho-regulated cell spreading was found to be the directly selected trait, with yes-associated protein 1 translocation to the nucleus mediating fitness on soft ECM. Overall, these data show that genetic variation can drive cancer cell adaptation to ECM stiffness.

## Significance

How changes to the stiffness of the extracellular matrix impact tumor progression is not fully understood. Here, we used the method of experimental evolution to ask how the considerable cell-to-cell genetic variation in cancer cells might interact with extracellular matrix (ECM) stiffness. Our results show the existence of rare tumor clones that are adapted to soft ECM, displaying highly unusual behaviors on soft ECM like spreading, traction force generation, increased growth rate, and nuclear localization of yes-associated protein 1 (YAP). These data show that genetic variation can drive cancer cell adaptation to ECM stiffness.

Tumors are genetically much more heterogeneous than the corresponding cells of their origin (1–5). How the tumor microenvironment interacts with the diverse genetic makeup of tumor cells, resulting in the emergence of increasingly malignant cells, is not well understood (6–8). One possibility is that the changing microenvironment favors specific clones that are better adapted to or “fit” for it, via natural selection. Such selection may also act on tumor cell populations that metastasize to secondary sites with entirely new microenvironmental niches compared with their tissue of origin (9–12). The specific properties of the tumor microenvironment that could impose evolutionary pressure, and the properties of the genetic variants that may be selected, are not clear.

Mechanical stiffness of the extracellular matrix (ECM) increases in many solid cancers and contributes to tumor progression, which is why many solid tumors are first detected by self-palpation as stiff lumps in the tissue (13–15). Also, mechanical stiffness of the ECM in secondary tumor sites of metastasis can be lower than the tissue of origin [e.g., solid breast tumors (~40 kPa) versus the brain niche (~1 kPa)] or higher [e.g., breast solid tumors (~40 kPa) versus the bone (~1 GPa)] (16–18). Mechanical stiffness can also be spatially distributed in a growing solid tumor; for example, stiffness may be low in the core of the tumor and high at the margins (19, 20). Such altered tumor stiffness can in turn reciprocally influence tumor cell behavior by promoting cancer cell proliferation (13), the loss of tissue structure (13, 15), invasive cancer cell migration (21, 22), and resistance to therapeutic drugs (23, 24). Further, the phenomenon of “mechanical memory” has been reported [reviewed by us (25)], where cells in an ECM with “new” stiffness can store and recall memory of having been on an ECM with a different stiffness (26–28).

**Fig. 1.**
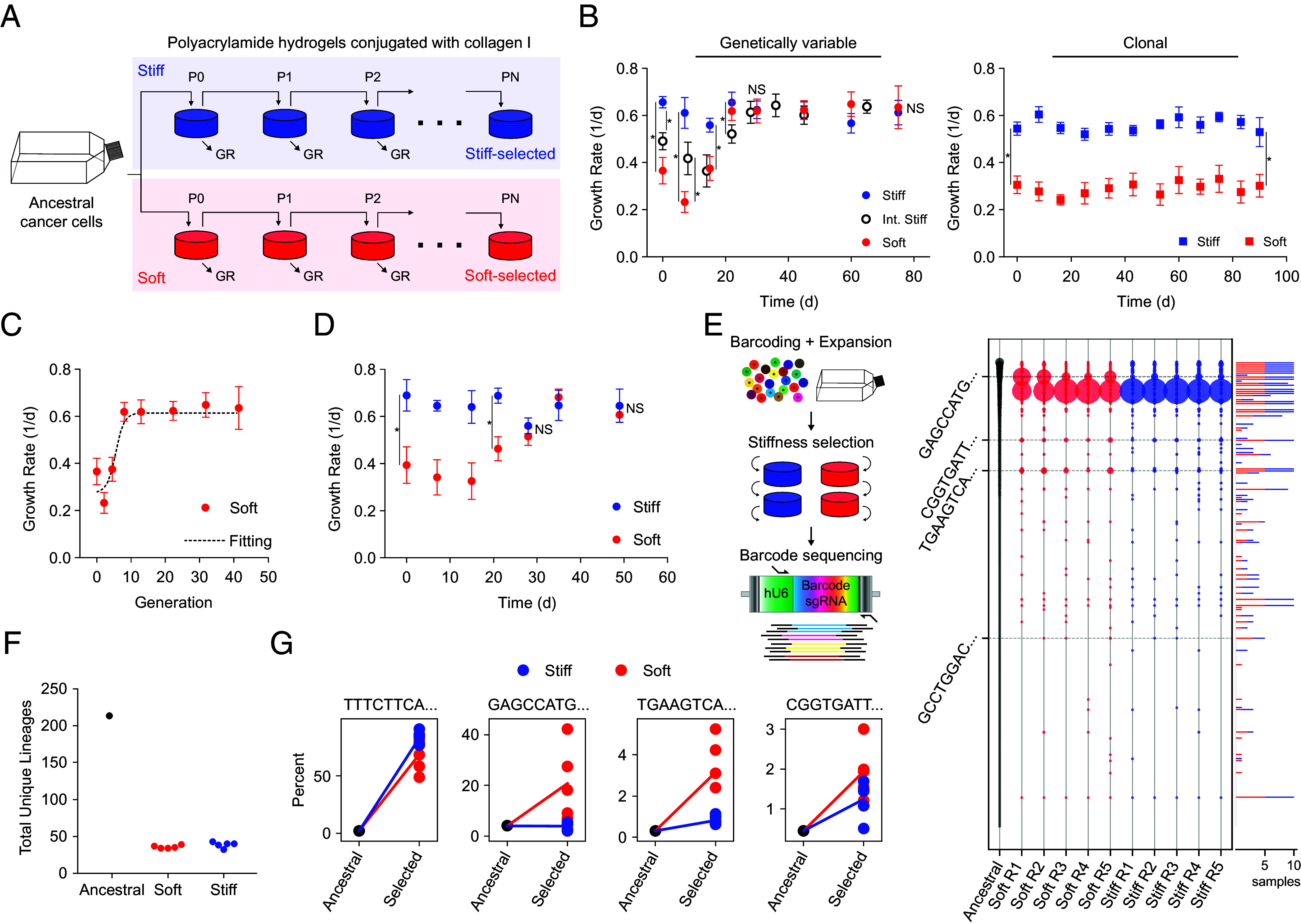
Evidence of cancer cell evolution on soft model ECMs. (*A*) Schematic of the evolution experiment. (*B*) MDA-MB-231 human breast carcinoma cells were cultured on collagen type I conjugated soft (red, *E* = 1 kPa), intermediate stiff (Int. stiff; hollow, *E* = 22 kPa), or stiff (blue, *E* = 308 kPa) hydrogels for up to 75 to 90 d. Mean growth rate (GR) is shown as a measure of fitness of selected lines measured at different times during the sustained culture in genetically variable ancestral populations (*Left*) or clonal lines (*Right*). Error bars, SEM (10 replicate lines on all stiffness). Statistically significant differences between growth rates at each time-point were determined by the Mann–Whitney *U* test, *P < 0.05; nonsignificant (NS): *P* > 0.05. (*C*) Nonlinear regression of equation from deterministic selection theory (dashed line) to the generation-dependent growth rate of populations (circles) measured on soft ECM in *B*. Error bars, SEM (10 replicates). (*D*) MDA-MB-231 cells labeled with heritable DNA barcodes underwent the same selection as in *A*. Mean growth rate is shown of barcoded MDA-MB-231 lines cultured on the stiff (blue) and soft (red) ECM, measured at multiple time points during sustained 7-wk culture. Error bars, SEM (five replicates on each stiffness). *P < 0.05; NS: *P* > 0.05 by the Mann–Whitney *U* test. (*E*) Abundance of barcoded MDA-MB-231 clones was identified with targeted amplification and sequencing of DNA barcode tags, shown in dot plot of sequences ordered by total abundance within the ancestral population. Dots are sized according to percent abundance. The bar plot to the right is the total number of stiffness-selected samples each lineage is found in. The four labeled barcodes have a higher average change in abundance from the ancestral population in the soft-selected cells compared to the stiff-selected cells. (*F*) Unique clonal barcode sequences (lineages) in the ancestral population (black), soft-selected group (red; five replicates), and stiff-selected group (blue; five replicates). (*G*) Percent total abundance of four clones with a positive average log_2_(fold change) on soft ECM. Dots represent percent abundances of individual replicates in the respective populations.

Prior studies on the role of ECM stiffness in cancer have largely not accounted for the well-known genetic heterogeneity of tumors. Individual genetic variants may possess a very different response to ECM stiffness from what is apparent in population-level studies such that ECM stiffness could select for the “fittest” variants. Such selection could have functional implications for tumor progression. Here, we performed sustained culture of genetically variable tumor cell populations and clonal populations on soft and stiff polyacrylamide hydrogels conjugated with collagen (Fig. 1*A*) to understand how ECM stiffness interacts with the heterogeneous genetic makeup of tumor cells.

## Results

### Evidence for Cancer Cell Evolution on Soft Model ECMs.

We performed studies with two different cancer cell lines on soft and stiff ECM: MDA-MB-231 human breast carcinoma cells and HT-1080 human fibrosarcoma cells. These cell lines have been maintained in culture on a stiff substrate (plastic tissue culture dishes) for decades and have typically accumulated genetic variation for approximately 10^4^ generations (29, 30). Ten replicate ancestral lines were initiated by plating 2 × 10^4^ cells on polyacrylamide gels of Young’s modulus E = 1 kPa (soft), 22 kPa (intermediate stiff), and 308 kPa (stiff) (Fig. 1*A*) conjugated with type I collagen (10 replicate lines for each stiffness). While questions have been raised in the past about polyacrylamide gels for their ability to control stiffness independently of ligand tethering and porosity (31), we and others have subsequently shown that cells indeed sense stiffness in the polyacrylamide gel system (32–34). Ancestral cell populations were allowed to grow for approximately 7 d, and then 2 × 10^4^ cells were passaged onto fresh substrates of the same (corresponding) stiffness (Fig. 1*A*; cell doubling times were roughly 24 to 48 h). This procedure was carried out for 75 d, i.e., for approximately 40 cell generations. Consistent with previous studies (35), the initial growth rate of cells on the soft ECM was approximately half that on the stiff ECM (Fig. 1 *B*, *Left*). After about 1 mo of sustained culture on the soft ECM, the growth rate was statistically indistinguishable from that on the stiff ECM and remained that way for the rest of the experiment (Fig. 1 *B*, *Left*). The growth rate in ancestral cells was lower but by a smaller amount on the intermediate stiff ECM as compared to the soft ECM and became similarly indistinguishable from the stiff ECM growth rate by 1 mo of culture. We repeated these experiments with HT-1080 cells and found similar results (*SI Appendix*, Fig. S1*A*). Importantly, the growth rate of genetically homogeneous cancer lines derived from 10 individual clonal cells did not increase even after culture for 3 mo on the soft ECM (Fig. 1 *B*, *Right*). The lack of adaptation in clonal cells on the soft ECM suggests that the observed increase in growth rate in nonclonal populations (Fig. 1 *B*, *Left*) required genetic heterogeneity. These data are consistent with the interpretation that the increase in growth rate or fitness in genetically variable populations occurred due to natural selection of genetic variants optimally adapted to the soft ECM. The lack of clonal cell fitness adaptation also argues against an explanation in which gradual adaptation of pathways that regulate growth rate eventually causes an increase in the mean growth rate of the population, as would occur in a mechanism involving “mechanical memory.” Interestingly, the observed adaptation was matrix-protein dependent because there was no change in growth rate even in genetically variable MDA-MB-231 cell populations when cultured on gels coated with fibronectin (*SI Appendix*, Fig. S1*B*). There was no difference in apoptosis between selected and ancestral cells on both stiff and soft ECM (*SI Appendix*, Fig. S2). This suggests that the observed growth rate advantage in the soft-selected cells is likely due to higher proliferation rather than lower apoptosis.

The observed difference in the growth rate of the ancestral population on stiff and soft ECM at day zero (d0) of culture, and its disappearance after sustained culture, implies that selected clones on soft ECM are likely present at low frequency in the starting ancestral population. To infer the approximate initial frequency and fitness advantages of the selected clones, deterministic selection theory applied to competing clones was employed (36). After *t* generations, the frequency of a focal clone, *p_t_*, can be calculated from the equation $\frac{p_{t}}{q_{t}}=\frac{p_{0}}{q_{0}}{\left(w^{*}\right)}^{t}$, where *q = 1 − p* represents the frequency of the competing clone(s); *p_0_* is the initial frequency of the focal clone; and w* is the fitness advantage of the focal clone relative to the wild-type. We estimated the fraction of variants in the ancestral population that are fit on the soft ECM by performing nonlinear least-squares fitting on the measured growth rate versus generation. The data were described well by selection theory (Fig. 1*C*), predicting an increase in the frequency of an initially rare clone or clones (*p_0_* = 1.03% of the ancestral population) within 10 generations, leading to an approximately twofold proliferation rate advantage over wildtype (w* = 2.23).

We attempted to more directly test the hypothesis that ECM stiffness selects for genetic variants in a population using a previously described lineage-tracing system, ClonMapper, to systematically track clonal abundance across a population of cells (37). We transduced MDA-MB-231 cells with a ClonMapper DNA-barcode library, based on a variant of the CROP-seq vector containing a blue fluorescent protein (BFP) reporter and random barcodes of 20 base pairs (bp) in length with no ambiguous (N) bases. Cells were transduced with a low multiplicity of infection (MOI = 0.1) to minimize multiple barcode integrations per cell. BFP-tagged cells were isolated using fluorescent activated cell sorting (FACS) and expanded to reach a starting diversity of ~450 unique barcodes, and subsequently archived for experimental evolution. We repeated the evolution experiment with five ancestral replicates each of 2 × 10^4^ barcoded MDA-MB-231 cells on soft and stiff ECM. Similar to the results in Fig. 1*B*, the growth rate of the barcoded population showed an adaptation after many days of sustained culture on soft ECM and remained indistinguishable from that on stiff ECM for up to 7 wk (Fig. 1*D*). Next, we carried out targeted sequencing of barcodes on the cells selected on hydrogels as well as the ancestral cells to assess clonal abundance (Fig. 1 *E*–*G*). ~220 unique barcodes were detected in the ancestral population, indicating a multiplicity of ~100× for an average starting frequency of 0.005. After selection on stiff and soft ECM, there was an ~80% decrease in the number of unique barcoded clones detected across all replicates (Fig. 1*F*) and a change in abundance distribution of surviving clones (Fig. 1*E*). Ten barcoded clones were detected in replicates of both ECM populations with increased log_2_(fold change) after selection (Fig. 1*G*; the top four clones are shown). One barcoded clone (TTTCTTCA…) had the highest fitness on both ECM, but whereas all five stiff replicates were nearly fixed for that lineage, four other clones showed a greater increase on soft ECM relative to stiff ECM (with the most abundant being GAGCCATG…), suggesting preferential selection on soft ECM (barcodes labeled in Fig. 1*E*). The barcoded clone that was selected equally on soft and stiff ECM is likely due to selection on type I collagen (independent of stiffness), while clones that are preferentially selected on one stiffness compared to the other, reflect variants optimally adapted to that stiffness. These results are further evidence that ECM or ECM stiffness can exert natural selection, and that genetically variable tumor cell populations can respond to that selection.

### Transcriptomic Differences in Soft-Selected and Ancestral Cells.

To explore the mechanism underlying evolution on soft ECM, we performed whole genome transcriptome RNA sequencing (RNA-seq). Principal component analysis (PCA) on the gene expression profiles of wildtype MDA-MB-231 cells for all the conditions showed that the major separation (PC1; 50.68% of variance explained) in the gene expression profiles exists between the ancestral and selected population irrespective of the stiffness. From the PCA, it was also evident that the next factor (PC2; 21.51% of variance explained) that contributed to differences in gene expression profiles of these samples is the matrix stiffness (*SI Appendix*, Fig. S3*A*). Soft-selected cells exhibited significant changes in gene expression compared to the ancestral population on soft ECM (*P_adjust_* < 0.01; up-regulated genes = 2,514, down-regulated genes = 2,530). Likewise, stiff-selected cells had significant changes in gene expression compared to the ancestral population cultured on stiff ECM (*P_adjust_* < 0.01; up-regulated genes = 2,061, down-regulated genes = 2,183; *SI Appendix*, Fig. S3*B*; see *Materials and Methods*). Gene ontology (GO) enrichment analysis on the differentially expressed genes in soft-selected cells showed that the genes significantly enriched in biological processes were related to cell adhesion and ECM organization pathways (*SI Appendix*, Fig. S3*C*; see *Materials and Methods*). While similar processes related to cell adhesion and ECM organization were also enriched in stiff-selected cells, the GO analysis of differentially expressed genes between soft-and stiff-selected cells still showed enrichment in cell adhesion (*SI Appendix*, Fig. S3*C*). This suggests differences in the adhesion ability of cells selected on different stiffness corresponding to separation along PC2 in *SI Appendix*, Fig. S3*A*. Collectively, these results show that there are genome-wide differences in gene expression between the enriched clones in the soft-selected population and the ancestral population. Further, differences in expression of genes related to cell adhesion may mediate the differences in fitness between soft-selected cells and ancestral cells on soft ECM.

### Soft-Selected Cells Spread, Assemble F-actin Fibers, and Exhibit Yes-Associated Protein 1 (YAP) in the Nucleus on Soft ECM.

**Fig. 2.**
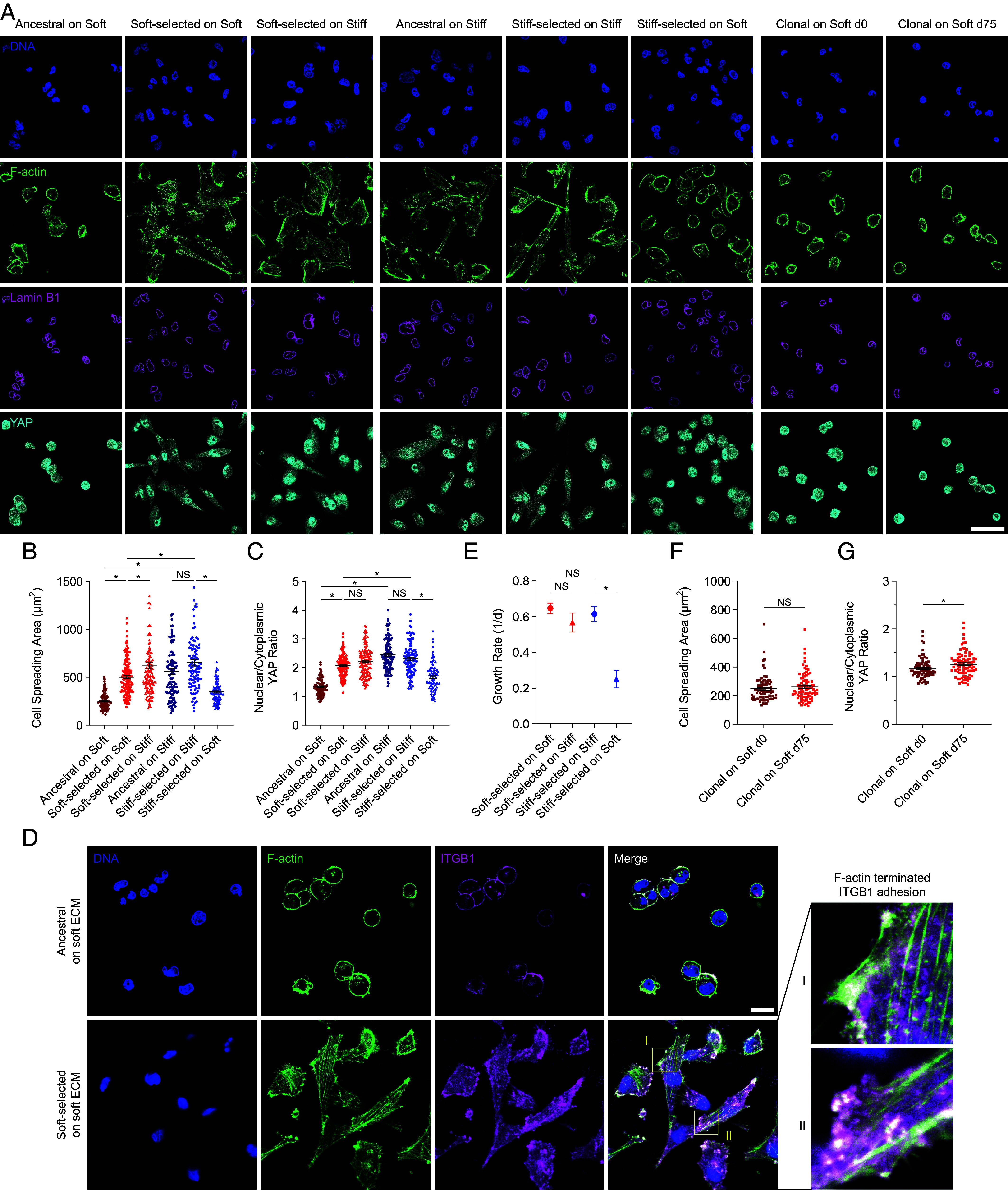
Soft-selected cells spread, assemble F-actin fibers, and exhibit YAP in the nucleus on soft ECM. (*A*) Representative images of DNA (blue), F-actin (green), lamin B1 (magenta), and YAP (cyan) in ancestral, soft- or stiff-selected, and clonal cells cultured on stiff or soft ECM (here, soft refers to 1 kPa, and stiff refers to 308 kPa gels). (Scale bar: 50 μm.) Corresponding quantification of cell spreading area (*B*) and nuclear to cytoplasmic YAP intensity ratio (*C*) is shown of ancestral cells and soft- or stiff-selected MDA-MB-231 cells cultured on stiff and soft ECM. Mean values are calculated from >100 cells from three replicate lines. Error bars, SEM. *P < 0.05; NS: *P* > 0.05 by ordinary one-way ANOVA. (*D*) Representative images of DNA (blue), F-actin (green), and β1-integrin (ITGB1; magenta) in ancestral and soft-selected cells both cultured on soft ECM. Enlarged views show F-actin fibers terminating in integrin-marked adhesions. (Scale bar: 50 μm.) (*E*) Mean growth rate is shown of selected populations after 8-wk selection on soft or stiff ECM, followed by culture on soft or stiff ECM. Error bars, SEM (six replicates on both stiffness). *P < 0.05; NS: *P* > 0.05 by ordinary one-way ANOVA. Quantification of cell spreading area (*F*) and nuclear to cytoplasmic YAP intensity ratio (*G*) in clonal cells cultured on soft ECM at day 0 and day 75. Mean values are calculated from >80 cells from three replicate lines. Error bars, SEM. *P < 0.05; NS: *P* > 0.05 by the Mann–Whitney *U* test.

Motivated by the genomic analysis that suggested significant differences in cell adhesion between soft-selected and ancestral cells on soft ECM, we investigated cancer cell phenotypes on soft and stiff ECM. As expected, ancestral cells were unable to spread on soft ECM, featured rounded, irregular lamin B1 stained nuclei, an absence of central filamentous actin (F-actin) stress fibers, and nuclear YAP (Fig. 2*A*). Remarkably, cells that had been cultured for 75 d on soft ECM exhibited spread morphologies, cortical F-actin and central F-actin fibers, flatter, less wrinkled nuclei, and nuclear YAP, even on soft ECM (quantification and statistical comparisons are shown in Fig. 2 *B* and *C*). Likewise, soft-selected cells assembled clear β1-integrin–containing adhesions on soft ECM, with visible F-actin fibers that appear to terminate into at least a subset of these adhesions (Fig. 2*D*). This demonstrates the unusual ability of soft-selected cells to polymerize F-actin, that, through interactions with integrins, will promote spreading even on soft ECM. Importantly, these soft-selected cells exhibited similar properties when cultured on stiff ECM, as on soft ECM. Conversely, cells cultured for 75 d on stiff ECM, exhibited similar properties as ancestral cells on stiff ECM (Fig. 2 *A*–*C*), but were unable to spread on soft ECM, and YAP was excluded from their nuclei. Consistent with these observations, the growth rate of soft-selected cells on soft ECM was indistinguishable from that on stiff ECM, while the growth rate of stiff-selected cells was significantly lower on soft ECM compared to stiff ECM (Fig. 2*E*). Both the degree of spreading and YAP nuclear localization were roughly comparable between soft-selected cells on soft ECM and stiff-selected cells on stiff ECM. Consistent with the lack of growth rate adaptation in clonal populations (Fig. 1*B*), clonal cells cultured for 90 d on soft ECM showed no difference in spreading nor YAP localization to the nucleus compared with clonal cells cultured at d0 on soft ECM (Fig. 2 *A*, *F*, and *G*).

### Rho-Regulated Cell Spreading Is the Directly Selected Trait while YAP Mediates Fitness on Soft ECM.

**Fig. 3.**
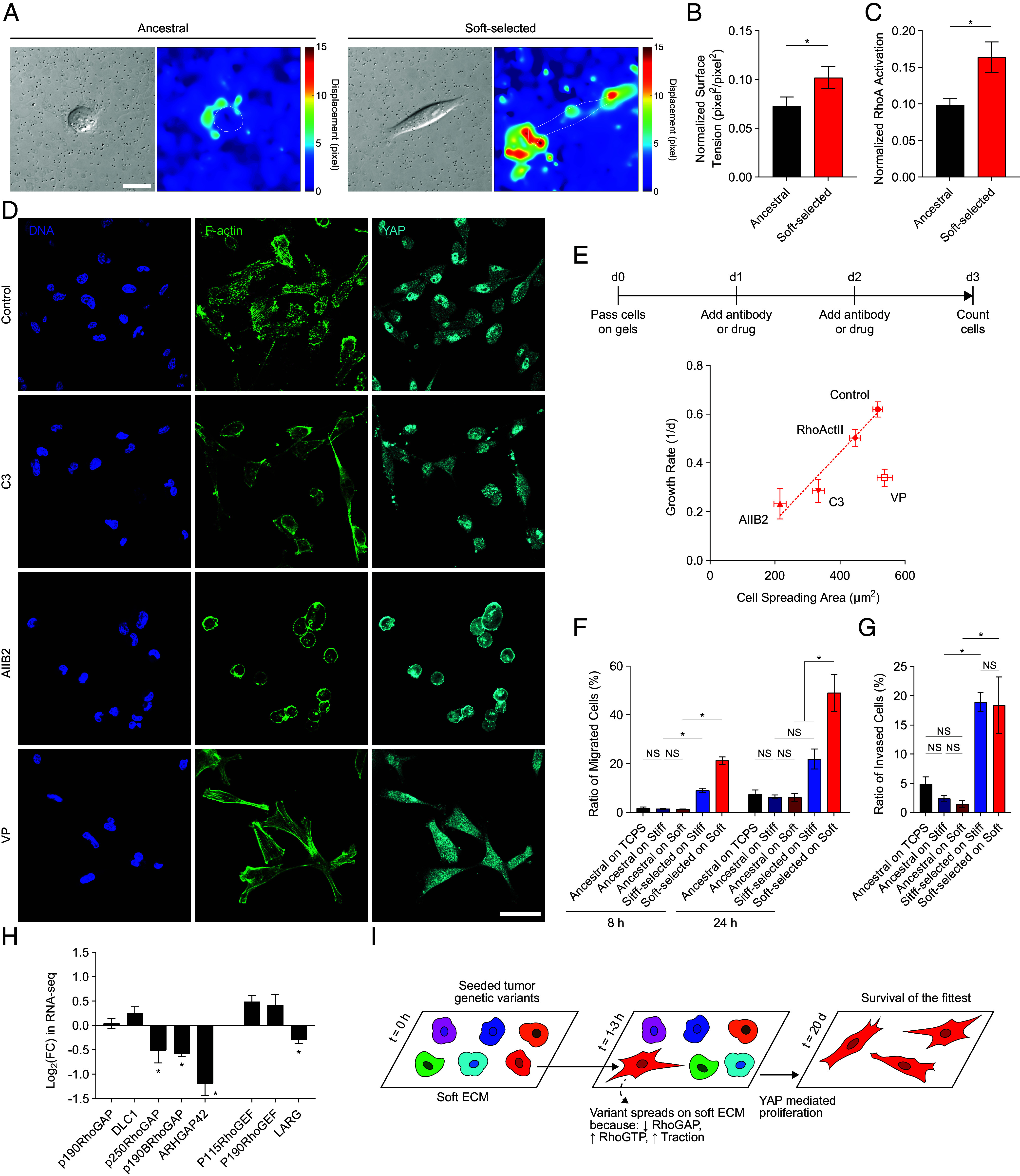
Rho-regulated cell spreading is the directly selected trait while YAP mediates fitness on soft ECM. (*A*) Representative differential interference contrast images and bead displacement heatmaps of ancestral and soft-selected cells cultured on soft ECM. (Scale bar: 20 μm.) (*B*) Normalized surface tension calculated from displacement field. Error bars, SEM (13 replicates). *P < 0.05 by the Mann–Whitney *U* test. (*C*) Normalized RhoA activation quantified using the G-LISA^®^ assay of ancestral and soft-selected cells cultured on tissue culture plastic. Error bars, SEM (data were collected from three replicate lines with two technical replicates per line). *P < 0.05 by the Mann–Whitney *U* test. (*D*) Representative images of DNA (blue), F-actin (green), and YAP (cyan) in soft-selected cells cultured on soft ECM under treatment with C3 transferase (C3), AIIB2 antibody, or verteporfin (VP). (Scale bar: 50 μm.) (*E*) Mean cell spreading area and mean growth rate in soft-selected cells cultured on soft ECM (control) under treatment with C3 transferase (C3), AIIB2 antibody, VP, or Rho activator II (RhoActII). Error bars, SEM. Mean spreading area is calculated from >80 cells in three replicate lines; growth rates were measured in at least four replicate lines. (*F*) Migration and (*G*) invasion of MDA-MB-231 cells were quantified using the Boyden chamber assay with a serum gradient. Error bars, SEM (10 replicate lines pooled into five groups for selected lines, and five biological replicates were analyzed for ancestral cells). *P < 0.05; NS: *P* > 0.05 by ordinary one-way ANOVA. (*H*) Fold change of RhoA GTPase-activating protein and Guanine nucleotide exchange factor gene expression in soft-selected cells cultured on soft ECM compared to ancestral cells cultured on soft ECM. Error bars, SEM (four replicates). *P < 0.05 by the Mann–Whitney *U* test. (*I*) The schematic diagram shows the selection mechanism for certain variants to spread and proliferate more on soft ECM.

The ability of soft-selected cells to spread on soft ECM is unusual because cells typically are unable to exert significant traction force on soft ECMs (stiffness of 1 kPa) (38). We cultured ancestral or soft-selected cells on collagen conjugated soft polyacrylamide gels containing embedded 0.5 μm fluorescent beads. Taking images of cells and fluorescent beads before and after treatment with the detergent sodium dodecyl sulfate (SDS) to remove cells, allowed us to quantify the relaxation of bead positions due to cell removal. We summed up the squared displacements of the beads and estimated a normalized surface tension following the approach of Odde and coworkers (39). As seen in Fig. 3*A*, the bead displacements were significantly higher in soft-selected cells cultured on soft ECM compared to ancestral cells, and the normalized surface tension was significantly higher in soft-selected cells (Fig. 3*B*). These results show that soft-selected cells spread on soft ECM by exerting abnormally high traction.

As F-actin stress fiber formation and traction force generation is regulated ultimately by RhoA signaling, we examined RhoA GTPase activity using a G-LISA^®^ assay (Cytoskeleton Inc.). Soft-selected cells possessed a higher level of RhoA activation compared to the ancestral population (Fig. 3*C*). Treating soft-selected cells with Rho kinase inhibitor C3 transferase, which stabilizes the inactive form of RhoA, decreased the degree of cell spreading and also growth rate (Fig. 3 *D* and *E*). Treating soft-selected cells with Rho activator II had no effect on spreading or growth rate, which is consistent with the high level of RhoGTP already present in these cells (Fig. 3*E*). To challenge the hypothesis that cell spreading drives proliferation on soft ECM, we inhibited β1 integrins by treating cells with anti-integrin β1 AIIB2 antibody which has been used previously to inhibit these integrins in MDA-MB-231 cells (40) and other cell types (41). Treatment of spread cells with the inhibitory antibody reduced the spreading of the soft-selected cells on soft ECM and abrogated the nuclear localization of YAP (Fig. 3*D*). Additionally, the growth rate decreased by nearly threefold on the soft ECM upon β1-integrin inhibition for 3 d compared to untreated cells (Fig. 3*E*). These results support the interpretation that RhoA-regulated cell spreading is the primary determinant of growth rate (i.e., fitness) in the selected cancer cell populations. That is, cell spreading is the directly selected trait that confers high fitness on soft-selected cells cultured on soft ECM.

Given that soft-selected cells feature YAP in the nucleus on soft ECM, we hypothesized that YAP mediates the effects of cell spreading on proliferation on soft ECM. Inhibiting YAP with verteporfin (VP) did not alter the spreading of soft-selected cells on soft ECM; yet, there was a significant decrease in cell proliferation (Fig. 3 *D* and *E*). We conclude that the YAP pathway mediates fitness in soft-selected cells on soft ECM.

### Selection on Soft ECM Results in Highly Migratory Cells.

We next examined the functional consequences of natural selection by soft ECM by quantifying the three-dimensional migration of soft-selected, stiff-selected, and ancestral cell populations through porous membranes in a Boyden chamber assay. Soft-selected populations (selected for 75 d) exhibited a several-fold increase in migration across the porous membrane under a serum gradient, compared to stiff-selected cells (selected for 75 d) or ancestral cells cultured for 3 d on different substrates (Fig. 3*F*). Soft-selected cancer cell clones are thus highly migratory compared with nonselected cells or cells selected on stiff ECM. We also examined the invasive capability of the cancer cells by quantifying the number of cells that passed through a Matrigel-coated porous membrane in the Boyden chamber (Fig. 3*G*). Consistent with the migration results, the soft-selected cells exhibited high invasion compared to ancestral cells.

## Discussion

It is well known that there is significant genetic heterogeneity from cells to cells in a developing solid tumor. The tumor microenvironment can select for specific genetic variants in the tumor, and the resulting evolution can dictate the fate of the tumor (42). Tumor evolution is important in the context of eventual metastatic spread and drug resistance (43); however, the factors that drive tumor evolution have not been fully elucidated (44). Here, we demonstrated the principle that ECM stiffness, a key property of the changing tumor microenvironment, can exert natural selection on genetically variable tumor cell populations. Using the well-established method of experimental evolution in which replicate lines of genetically variable cell populations were exposed to a novel selection pressure of a soft ECM, we found that the soft ECM consistently caused an increase in fitness of the population, over time scales of a few weeks. That this increase in fitness is because of a selection operating at the genetic level is evident from the observation that replicate clonal cell lines do not respond to selection pressure by the soft ECM. Also, barcoding experiments revealed a clear and consistent enrichment of specific clones across different experiments, providing direct evidence of selection at the genetic level.

The finding that some genetic variants in the population proliferate as much on the soft ECM as stiff ECM is surprising given the widely reported observation that tumor cells proliferate more on stiff ECM (13, 38, 45–49). Consistent with these other studies, in our experiments, cancer cells proliferated twice as fast on the stiff ECM as soft ECM in the initial period of culture (e.g., for the first 1 wk or so). Over longer periods of time –2 to 3 wk and beyond—clones that proliferate well on soft ECM became enriched in the population resulting in a gradual increase in the population-level proliferation on soft ECM. These findings highlight the fact that without a sustained culture-type experiment, properties of rare clones can be missed in studies. Importantly, these clones proliferated well not only on the soft ECM but also on the stiff ECM (Fig. 2*E*), indicating that they are distinct in properties from cancer cell populations that proliferate well on stiff ECM relative to soft ECM. Thus, our studies highlight the importance of studying the responses of individual genetic variants in cancer cell populations to ECM stiffness.

The directly selected trait on which natural selection by soft ECM acts was found to be Rho-regulated cell spreading. Selection of tumor cell populations on soft ECM resulted in populations with a propensity for migration and proliferation, properties that are well appreciated to be important clinically in tumor progression. Soft-selected clones exhibited significant and consistent differences in genome-wide gene expression compared to ancestral populations. The genomic evidence is consistent with natural selection that results in enrichment of genetic variants on soft ECM; the genetic differences would be expected to manifest in systematic changes in gene expression patterns on a given ECM stiffness.

Soft-selected cells were able to spread on soft ECM at levels comparable to normal cell spreading on stiff ECM, assemble integrin containing adhesions, and generate high traction. These behaviors are remarkable because cells are typically unable to spread on soft ECM (~1 kPa Young’s modulus), and this is indeed reflected in the population level low mean spreading area in cancer cells at d0. Spreading of soft-selected cells on soft ECM is clearly driven by high Rho levels, high traction force and the ability to polymerize F-actin and assemble integrin-containing adhesions. Selection occurs on the trait of cell spreading because inhibiting spreading resulted in predictable changes in growth rate (50). The high Rho GTPase activity in soft-selected cells can be explained by low levels of Rho GTPase activating proteins (RhoGAPs) in soft-selected cells relative to ancestral cells with more than a twofold decrease in ARHGAP42 levels (Fig. 3*H*). The low levels of ARHGAP42 correlated with a general decrease in DNA methylation of two regions in the ARHGAP42 gene body (*SI Appendix*, Fig. S4), which is known to correspond to lower expression (51, 52); the promoter methylation was unaltered. Importantly, inhibiting YAP in soft-selected cells did not affect spreading on soft ECM but reduced the growth rate significantly, suggesting that the YAP pathway mediates fitness conferred by spreading on soft-selected cells on soft ECM. These results collectively suggest the selection mechanism outlined in Fig. 3*I*.

Transferring soft-selected cells to stiff ECM revealed that they continued to proliferate well on the stiff ECM. And yet, our deterministic theory suggests that they are present at low levels in the ancestral population (which was propagated for many generations on hard surfaces), which would require a low growth rate on the stiff ECM, as we have recently reported in evolving populations of fibroblasts (53). One possibility is that there is frequency-dependent selection, whereby the slow-growing soft-selected genotypes have a survival advantage once the population approaches confluence that maintains them in the population at a low equilibrium frequency. Alternatively, this discrepancy may perhaps be explained by the process of mechanical memory, in which molecular pathways are proposed to store and recall “information” related to the mechanical stiffness of the ECM. Nevertheless, the process of natural selection that we propose to drive the increase in the overall fitness in the cancer cell lines on soft ECM is distinct from the process of mechanical memory. In the process of evolution, there is a selection at the genetic level of fit clones, and not all genetic clones will respond the same way.

Clones selected on soft ECM exhibited aggressive migration compared to ancestral cells, as well as cells selected on stiff ECM. We speculate that such selection may occur in vivo in the core of the growing tumor where there are fewer collagen fibers and a softer overall microenvironment. Such selection would then result in clones that not only proliferate in soft environments but are also aggressive in migration over time. The phenotypic measurements indicative of malignancy are supported by results from Gene Set Enrichment Analysis (GSEA), which show that genes with decreased expression in soft-selected cells were negatively enriched in oncogenic gene set signatures, such as genes up-regulated in primary epithelial breast cancer cells by overexpression of beta-catenin [CTNNB1, Normalized Enrichment Score (NES): −1.75], genes up-regulated by ATF2 (NES: −1.65) and genes up-regulated in breast cancer cell line MCF7 by overexpression of activated MEK1 protein kinase (MAP2K1, NES: −1.54) (*SI Appendix*, Fig. S3*D*). Likewise, genes with increased expression were enriched in oncogenic gene set signatures, such as genes down-regulated by knockdown of ALK (NES: 1.4), genes up-regulated by expression of oncogenic KRAS (NES: 1.37), and genes down-regulated by overexpression of SRC (NES:1.33). Additionally, soft-selected clones were able to not only spread and proliferate on soft ECM but also on stiff ECM, suggesting that these cells could then populate secondary sites independently of whether the new sites are soft or stiff.

In summary, our data provide evidence of an interaction between the stiffness of the ECM and genetic heterogeneity in tumor cell populations. Using the methods of experimental evolution, our data show that ECM stiffness is an agent of natural selection, which acts on cell spreading as the directly selected trait. Soft- selected cells are highly migratory. Our data raise the possibility that stiffness variations in solid tumors and in secondary sites may select for malignant population behaviors. Understanding the genetic underpinnings of the observed behaviors is a challenging task, and is a worthy future goal for gaining a full understanding of tumor evolution in the changing tumor microenvironment.

## Materials and Methods

### Synthesis and Functionalization of Hydrogels.

Polyacrylamide hydrogels were prepared using a well-established protocol (54). Acrylamide and bis-acrylamide (Bio-Rad) were mixed in 50:1, 40:1, and 12.5:1 ratios to prepare gels with Young’s modulus (E) of 1, 22, and 308 kPa, respectively, as previously described (33). The gel solution was degassed and mixed with 0.5% v/v ammonium persulfate (ThermoFisher Scientific) and 0.1% v/v tetramethylethylenediamine (ThermoFisher Scientific) to initiate polymerization. A volume of 100 μL of mixture per gel was sandwiched between a hydrophobic glass surface and a hydrophilic 18-mm diameter glass coverslip and polymerized for 20 min at room temperature. The gels were functionalized using sulfosuccinimidyl 6-(40-azido-20-nitophenylamino) hexanoate (G-Biosciences) and coated with rat tail collagen type I (0.2 mg/mL; Corning) before cell seeding.

### Cell Culture and Drug Treatment.

Cells were maintained in a humidified incubator at 37 °C and 5% CO_2_. Human breast carcinoma cells MDA-MB-231 [American Type Culture Collection (ATCC)] were cultured in Dulbecco’s Modified Eagle’s Medium with 4.5 g/L glucose (Corning), supplemented with 10% v/v donor bovine serum (Gibco) and 1% v/v penicillin/streptomycin (Corning). Human fibrosarcoma cells HT-1080 (ATCC) were maintained in Dulbecco’s Modified Eagle’s Medium with 4.5 g/L glucose (Corning), supplemented with 10% v/v fetal bovine serum (Gibco) and 1% v/v penicillin/streptomycin (Corning). Cells were passaged after reaching ~80% confluency (~every 5 to 7 d) by detaching cells using 0.25% trypsin (Corning) and replating onto new gels. For Rho disruption and YAP inhibition experiments, the cells were seeded on substrates and incubated overnight, followed by a replacement of media containing C3 Transferase (Cytoskeleton) at final concentrations of 1 μg/mL, Rho activator II (Cytoskeleton) at 1 μg/mL, or VP (MedChemExpress) at 10 μM. The treatment media were replaced every 24 h.

### Cloning.

MDA-MB-231 cells were seeded on a 10-cm diameter tissue culture dish at 500 cells per dish density. Following 14 d of incubation, the cells formed distinct colonies separate from one other. Each colony was isolated by placing an 8-mm diameter glass cloning ring (ThermoFisher Scientific) onto the dish surface that enclosed a single colony and trypsinizing the cells within the enclosed area (55). The colonies were then expanded in individual wells of tissue culture plate to obtain clones.

### Growth Rate and Apoptosis Measurements.

Growth rates of cell lines proliferated on gels were measured at specific time points. Cells were trypsinized from a substrate with a specified stiffness, and a set number of cells were seeded onto a new substrate with the same stiffness and allowed to grow for 3 d. On day 3, the cells were trypsinized from the gel surface and suspended in 750 μL of the cell culture medium. A volume of 10 μL of cell suspension was mixed with 0.4% trypan blue solution (ThermoFisher Scientific), and the live cell number was counted using the Countess 3 Automated Cell Counter (ThermoFisher Scientific) according to the manufacturer’s protocol. The growth rate was calculated as N_f_ = N_0_2^ηt^, where N_f_ is the final cell count; N_0_ is the initial cell count, which was determined by the same procedure before seeding and was fixed at 20,000 and 10,000 for MDA-MB-231 and HT-1080 cells, respectively; t is the time of 3 d; and η is the growth rate per day. To quantify apoptosis, cells were treated with 6 μM of CellEvent Caspase-3/7 Green Detection Reagent (Invitrogen) solution in phosphate-buffered saline (PBS) with 5% FBS for 30 min at 37 °C. The fluorescence signal was detected at Ex/Em = 502/530 nm using BioTek Cytation 5 (Agilent). Wild-type MDA-MB-231 cells cultured on tissue culture polystyrene were treated with 1 μM of staurosporine (Santa Cruz Biotechnology) for 6 h before measurement as a positive control.

### Generating Barcoded Cell Lines.

ClonMapper lentivirus was generated as described in the previous literature (56). Briefly, a high-complexity barcode insert, BgL-BsmBI, was generated in an extension reaction with a 60-bp oligonucleotide containing a 20-bp random sequence, CROPseq-PrimeF-BgL-BsmBI (GAGCCTCGTCTCCCACCGNNNNNNNNNNNNNNNNNNNNGTTTTGAGACGCATGCTGCA), and a reverse extension primer, CROPseq-RevExt-BgL-BsmBI (TGCAGCATGCGTCTCAAAAC), and then purified. BgL-BsmBI was then inserted into Cropseq-BFP-WPRE-TS-hU6-BsmbI (Addgene, 137993), using BsmBI (New England Biolabs, R0739S) and an overnight golden gate reaction at a backbone to insert molar ratio of 1:5. Assembled ClonMapper-N20-BFP plasmid was purified and concentrated and then transformed into electrocompetent SURE 2 cells (Agilent, 200152). Transformants were inoculated into 500 ml of 2×Yeast Extract Tryptone Medium containing 100 μg/mL carbenicillin and incubated overnight at 37 °C. Bacterial cells were pelleted, and plasmid DNA was extracted using a QIAGEN Plasmid Plus Midi kit (QIAGEN). ClonMapper-N20-BFP, PsPax2 (Addgene), and vesicular stomatitis virus glycoprotein G (Addgene) were transfected into HEK293T cells using Lipofectamine 2000 (ThermoFisher Scientific). Fresh medium was exchanged at 24 h. Viral particles were collected at 48 h posttransfection, filtered, and concentrated. MDA-MB-231 cells were seeded at 6 × 10^5^ per well in a six-well plate. After 24 h, cells were transduced with ClonMapper-N20-BFP lentivirus using 8 μg/mL polybrene for 16 h before removing virus and adding fresh culture medium. To reduce the likelihood of multiple barcode integrations per cell, the MOI was kept below 0.1. Forty-eight hours after transduction, 1,000 BFP-positive cells were isolated by FACS. Barcoded-MDA-MB-231 cells were expanded to 5 × 10^6^ cells with a measured diversity of 450 unique barcodes and cryopreserved in vials of 1 × 10^6^ cells each.

### Targeted Sequencing of Barcodes.

ClonMapper barcode sequences were amplified from genomic DNA as described in the previous literature (56). Briefly, genomic DNA was extracted for ancestral and selected populations using the PureLink Genomic DNA (gDNA) Mini Kit (ThermoFisher Scientific). To sequence barcoded populations, 2 μg of gDNA was loaded into a PCR and amplified with primers containing Illumina adapters and sequences flanking the N20 barcode for 25 cycles. All reactions were purified using a 1.6× to 0.7× Ampure XP bead cleanup (Beckman Coulter) and sequenced on an Illumina NextSeq 550 by paired-end sequencing for 2 × 75 cycles. Illumina reads were processed using a custom barcode extraction and processing pipeline, pycashier (57). Briefly, paired-end reads were merged into consensus single reads and quality-filtered with a Phred of 30, followed by barcode sequence extraction with an error tolerance of 0.1. To compensate for amplification artifacts and sequencing error, barcode sequences were clustered using starcode with a Levenshtein distance of 1 and minimum radius size of 3. Individual barcodes were then filtered out if not passing one of the given criteria: 1) >0.1% and found in the ancestral population, 2) in both soft-selected and stiff-selected samples or in more than two samples of single selected population, or 3) in one gel-selected sample >0.5% of the total population.

### RNA Sequencing.

Total RNA was extracted with the Direct-zolTM RNA MiniPrep Plus Kit (Zymo Research Corp). Library preparation was performed by the University of Florida Interdisciplinary Center for Biotechnology Research using an Illumina RNAseq library prep kit with ribodepletion. Sequencing was performed on a NovaSeq6000 S4 with a 2 × 150 bp read length kit to achieve approximately 100 million reads per sample (Illumina). RNA-seq data were processed and analyzed as described in the previous publication (58). Identification of differentially expressed genes and GO enrichment analysis were also performed as described in the previous publication (58). Oncogenic gene signatures were identified using GSEA v4.1.0 and the C6 MSigDB collection.

### Reduced Representation Bisulfite Sequencing (RRBS).

We applied RRBS to evaluate cytosine—phosphate—guanine (CpG) DNA methylation genome-wide (59, 60). RRBS utilizes a restriction enzyme that specifically cuts DNA at CCGG sites, allowing for targeted sequencing of regions enriched in CpG islands and regulatory elements. Subsequent bisulfite conversion converts unmethylated cytosines to uracils while preserving methylated cytosines, enabling the distinction between methylated and unmethylated CpG sites during sequencing. This approach provides a comprehensive and high-resolution assessment of DNA methylation patterns across the genome. Libraries were prepared from genomic DNA using the Ovation RRBS Methyl-Seq Kit (Tecan Trading AG) at the MD Anderson Cancer Center (MDACC) Epigenomics Profiling Core. In brief, 100 ng of genomic DNA was digested with MspI restriction enzyme, and Illumina-compatible cytosine-methylated adaptor was ligated to the enzyme-digested DNA. Following repair and bisulfite conversion, library preparation was done by PCR amplification. Libraries were quantitated using the Qubit 4 Fluorometer (ThermoFisher Scientific) and further evaluated using a 2100 Bioanalyzer Instrument (Agilent). Pooled libraries were sequenced on an Illumina NovaSeq 6000 instrument as 75 bp single reads at the MDACC Advanced Technology Genomics Core.

### RRBS Data Analysis.

The TrimGalore package was used to filter out reads with a phred33 quality score below 30 (61). Adapter trimming was performed, and reads shorter than 20 base pairs were discarded. The trimmed reads were aligned to the reference genome (hg38) using the Bismark tool (version 0.23.1) (62). Bismark performs alignment by incorporating bisulfite conversion information to accurately align reads to the appropriate genomic positions. Methylation information was extracted from the aligned reads using Bismark, producing a file containing the cytosine methylation calls for each genomic position. The edgeR package (63, 64) (version 3.36.0) in R (version 4.1.3) was employed for the statistical analysis of the differential methylation data. The methylation calls obtained from Bismark were processed to generate a count matrix, where each row represented a genomic feature (e.g., CpG site or region), and each column represented a sample. The count matrix contained the number of methylated and unmethylated reads for each genomic feature in each sample. Additionally, any necessary normalization steps, such as library size normalization or batch effect correction, were performed to account for technical variations and ensure comparability across samples. Genomic loci with low coverage or low variability across samples were filtered out to focus on robustly methylated regions in either of the conditions. The genomic loci that had read coverage of less than 25 reads were filtered out. Regions with at least 50% methylation in the majority of replicates of any condition (>75%) were included for the downstream analysis. In order to allow for a more comprehensive analysis of methylation patterns across more significant genomic regions, individual CpG loci within 200 bp were aggregated to create methylation regions. Methylated regions provide a broader view of the methylation landscape compared to individual methylated sites. By considering neighboring sites together as a region, it allows for the examination of local methylation patterns and potential regulatory elements or functional regions associated with the observed methylation. A summary count matrix of the methylation levels within each methylation region was generated by adding the methylation levels of the constituent CpG loci. The count matrix was normalized to account for differences in library sizes and sequencing depths between samples. Normalization factors were calculated using the edgeR package. A generalized linear model was constructed to model the relationship between methylation and the biological condition. A likelihood ratio test was performed to identify differentially methylated regions (DMRs) between interest groups. DMRs were determined based on significance (*P_adjust_* ≦ 0.01, and *P*-value ≦ 0.05).

### Immunofluorescence Staining and Microscopy.

Cells cultured on gels were fixed in 4% paraformaldehyde (Alfa Aesar) for 20 min at room temperature. Then, the fixed cells were permeabilized with 0.1% Triton X-100 (ThermoFisher Scientific) in PBS and blocked with 1 mg/mL bovine serum albumin (ThermoFisher Scientific) for 1 h at room temperature. Cells were incubated with primary antibodies mouse anti-YAP (sc-101199; Santa Cruz Biotechnology; dilution 1:100), rabbit anti-Lamin B1 (ab229025; Abcam; dilution 1:1,000), and rat anti-integrin β1 (MABT409 clone AIIB2; EMD Millipore; dilution 1:500) overnight at 4 °C. The samples were then washed with PBS and incubated with secondary antibodies Alexa Fluor 594 goat anti-rabbit (A11012; Invitrogen; dilution 1:200), Alexa Fluor 647 goat anti-mouse (A21235; Invitrogen; dilution 1:200), and Alexa Fluor 594 goat anti-rat (ab150168; Abcam; dilution 1:400) for 2 h at room temperature. Hoechst 33342 (Sigma-Aldrich) was used to stain DNA. F-actin was stained using Alexa Fluor 488 Phalloidin (Invitrogen; dilution 1:400). The samples were mounted on FluoroDish (World Precision Instruments) and imaged on an Olympus FV3000 confocal microscope using 60 × /1.3NA oil-immersion objective (Olympus Scientific Solutions Americas Corp.).

### Phenotype Quantification.

Images of F-actin shown by phalloidin staining were acquired at different locations on the hydrogel surface, and the cell boundary was manually labeled using ImageJ software to determine the cell spreading area. The nuclear boundary was traced in the DNA images. The averages of nuclear and cytoplasmic YAP intensities were quantified using the ImageJ measure tool. The nuclear to cytoplasmic YAP ratio was calculated as [(Nuclear intensity) ─ (Background intensity)] / [(Cytoplasmic intensity) ─ (Background intensity)].

### Transwell Migration and Invasion.

Cell migration and invasion were quantified using the Boyden chamber assay as described in the previous literature (65, 66). Cells cultured on hydrogels were serum-starved for 24 h and seeded at 2 × 10^5^ cells/cm^2^ density on top of a 24-well Transwell insert (Corning) with 8-μm pore size. For the invasion assay, 30 μL of Matrigel diluted with sterile ice-cold deionized water (1:2) was coated on the membrane for 1 h at 37 °C before cell seeding. Medium supplemented with 0.5% and 10% FBS was added to the top and bottom of the membrane respectively to establish a chemoattractant gradient. After 8 or 24 h of incubation at 37 °C and 5% CO_2_, the nonmigrated cells were removed from the upper membrane surface with a cotton swab, and the migrated cells were fixed and stained with Hoechst 33342. Membranes were then removed from the Transwell inserts with a scalpel and mounted onto slides where five fields of quintuplicate membranes were counted.

### Traction Force Microscopy.

Traction force microscopy for quantifying surface tension was performed as described previously (67). Red fluorescent microspheres (0.5 µm diameter; ThermoFisher Scientific) as fiducial markers were suspended in the polyacrylamide hydrogels fabricated as described in the above section. Cells were seeded at a low concentration (2,000 cells per gel) and incubated for 24 h at 37 °C and 5% CO_2_. Differential interference contrast and fluorescent images of isolated cells were taken simultaneously before and after treatment with 5% SDS (Sigma-Aldrich) solution. Traction force analysis was carried out using a customized MATLAB program and methods described by Chan et al. (39).

### Rho Activation Measurement.

The G-LISA^®^ RhoA Activation Assay Biochem Kit (luminescence based; Cytoskeleton) was used to quantify the RhoA activation in cancer cells. The experiment was performed following the manufacturer’s protocol, briefly described as follows. The cells were washed with ice-cold PBS and lysed using the lysis buffer on ice. After the harvest of the lysates with a cell scraper, the supernatant was collected. A volume of 10 µL of the lysate was isolated to measure protein concentration using Precision Red Advanced Protein Assay Reagent (Cytoskeleton) and BioTek Cytation 5 (Agilent). For the assay, the lysate was equalized to a protein concentration of 1 mg/mL and mixed with the binding buffer before being added into the reconstituted Rho affinity plate on ice. The lysis buffer and Rho control protein solution were also used as the blank and positive control, respectively. After the incubation on an orbital microplate shaker (400 rpm; ThermoFisher Scientific) at 4 °C for 30 min, the Rho affinity plate was sequentially treated with the antigen-presenting buffer, anti-RhoA primary antibody, and secondary horseradish peroxidase (HRP) labeled antibody. After adding the HRP detection reagent into the affinity plate, the luminescence signal was immediately detected using BioTek Cytation 5 (Agilent). The normalized RhoA activation is determined by dividing the signal values obtained from cell lysates by the positive control protein values.

### Statistical Analysis.

Nonlinear regression on deterministic selection theory equations was performed using Optimization Toolbox in MATLAB version R2022b (MathWorks). GraphPad Prism 10.0 was used for statistical analysis and graphic representations of data. Statistical tests included the Brown–Forsythe and Welch ANOVA test and Mann–Whitney test. Differences between values were considered statistically significant when *P* < 0.05 and nonsignificant for *P* > 0.05. The details of experimental conditions and statistical tests are provided in figure legends.

## Supplementary Material

Appendix 01 (PDF)

## Data Availability

The data reported in this paper have been deposited in the Gene Expression Omnibus (GEO) database under accession number GSE255829 (68). All other data are included in the article and/or *SI Appendix*.
