# Supplementary material for: Genetic variation drives cancer cell adaptation to ECM stiffness
Source: Proc Natl Acad Sci U S A. 2024 Sep 20;121(39):e2403062121. doi: 10.1073/pnas.2403062121 (PMC11441511; doi:10.1073/pnas.2403062121)
Supplement: Supplementary file 1 — Appendix 01 (PDF) [file pnas.2403062121.sapp.pdf]

PNAS

Supporting Information for
Genetic Variation Drives Cancer Cell Adaptation to ECM Stiffness

Ting-Ching Wang[a], Suchitaa Sawhney[b], Daylin Morgan[c], Richard L. Bennett[d], Richa Rashmi[e], Marcos R. Estecio[f], Amy Brock[c], Irtisha Singh[b,e], Charles F. Baer[g], Jonathan D. Licht[d], Tanmay P. Lele[a,b,h,*]
[a] Artie McFerrin Department of Chemical Engineering, Texas A&M University, College Station, TX 77843
[b] Department of Biomedical Engineering, Texas A&M University, College Station, TX 77843
[c] Department of Biomedical Engineering, The University of Texas at Austin, Austin, TX 78712
[d] Division of Hematology and Oncology, University of Florida Health Cancer Center, Gainesville, FL 32610
[e] Department of Cell Biology and Genetics, Texas A&M University, Bryan, TX 77807
[f] Department of Epigenetics and Molecular Carcinogenesis, The University of Texas MD Anderson Cancer Center, Houston, TX 77030
[g] Department of Biology, University of Florida, Gainesville, FL 32611
[h] Department of Translational Medical Sciences, Texas A&M University, Houston, TX 77030

* Correspondence: Tanmay P. Lele

Email: tanmay.lele@tamu.edu

This PDF file includes:

Figures S1 to S4

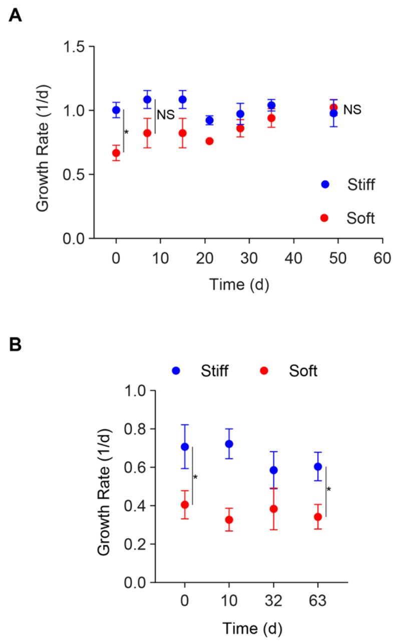

Fig. S1. (A) Mean growth rate is shown of genetically variable HT-1080 cells cultured on stiff (blue) and soft (red) ECM conjugated with collagen type I, measured at multiple time points during sustained 7-week culture. Error bars, standard error of the mean (SEM) (ten replicate lines on each stiffness). *$p < 0.05$; NS: $p > 0.05$ by Mann-Whitney U test. **(B)** Mean growth rate is shown of genetically variable MDA-MB-231 cells cultured on the stiff (blue) and soft (red) ECM conjugated with fibronectin, measured at multiple time points during sustained 9-week culture. Error bars, SEM (six replicate lines on both stiffness). *$p < 0.05$ by Mann-Whitney U test.

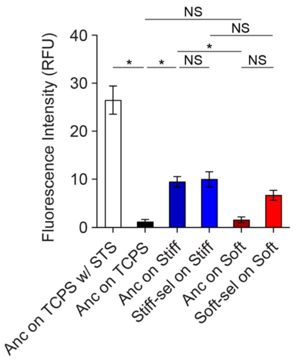

Fig. S2. Apoptosis was quantified by treating cells with CellEvent Caspase-3/7 Green Detection Reagent and measuring fluorescence signal at Ex/Em = 502/530 nm. Ancestral MDA-MB-231 cells cultured on TCPS were treated with 1 μM of staurosporine (STS) as a positive control. Error bars, SEM (ten replicate lines pooled into four groups for selected lines; and four biological replicates were analyzed for ancestral cells). *$p < 0.05$; NS: $p > 0.05$ by ordinary one-way ANOVA.

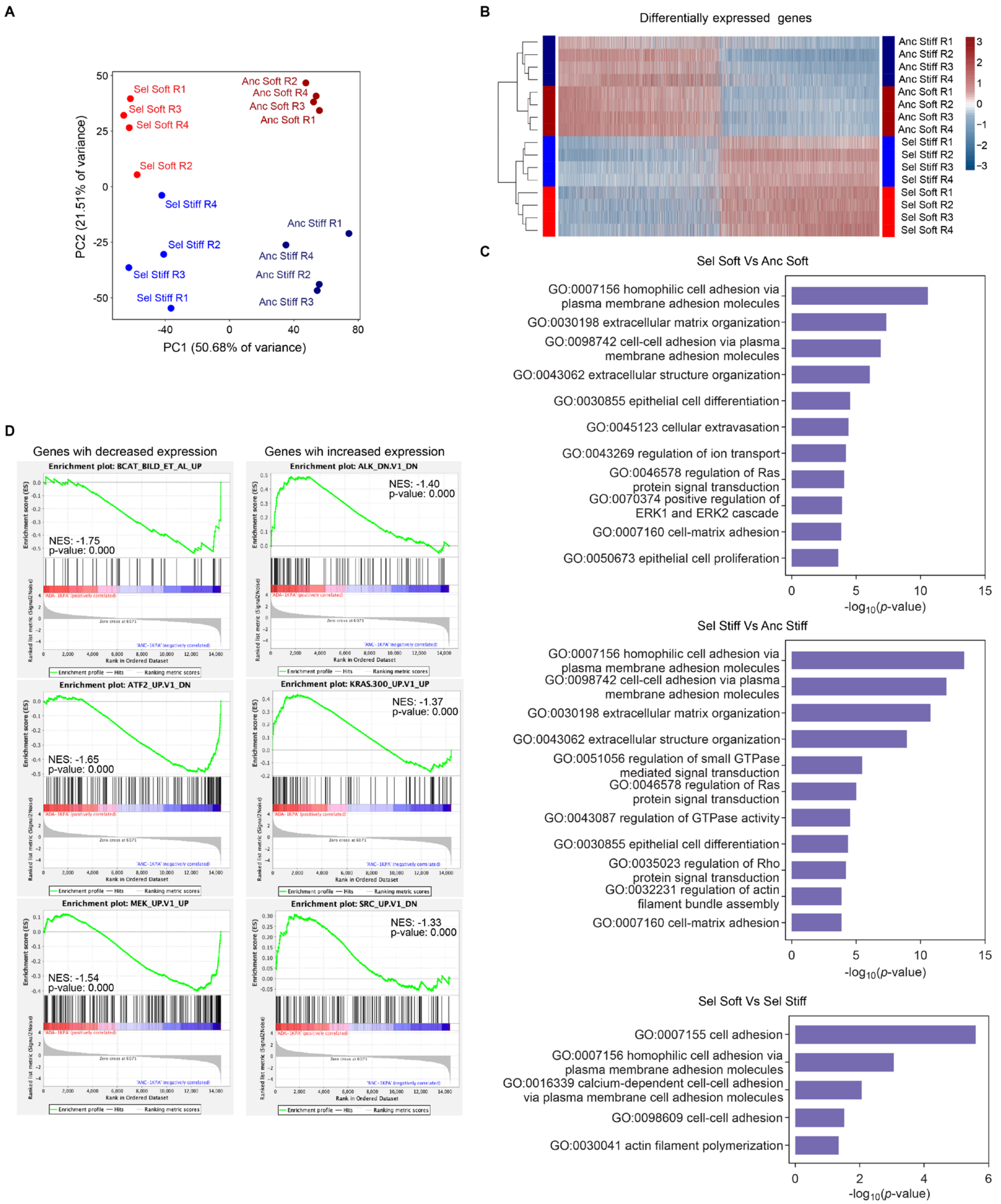

Fig. S3. (A) Principal component analysis (PCA) of gene expression (FPKM) using top 50% most variable genes by median absolute deviation (n = 6,291) of all expression genes. **(B)** Hierarchical clustering using FPKM z-score of all the differentially expressed genes (p_{adjust} < 0.01; n = 6,141). **(C)** Selected biological processes that are significantly enriched (p < 0.05) in soft-selected cells on soft ECM compared to ancestral cells on soft ECM (top), in stiff-selected cells on stiff ECM compared to ancestral cells on stiff ECM (middle), or in soft-selected cells on soft ECM compared to stiff-selected cells on stiff ECM (bottom). **(D)** Gene set enrichment analysis (GSEA) was performed using oncogenic gene set signatures. The top 3 gene sets for genes with decreased expression (left) or increased expression (right) in soft-selected populations are shown. NES is normalized enrichment score.

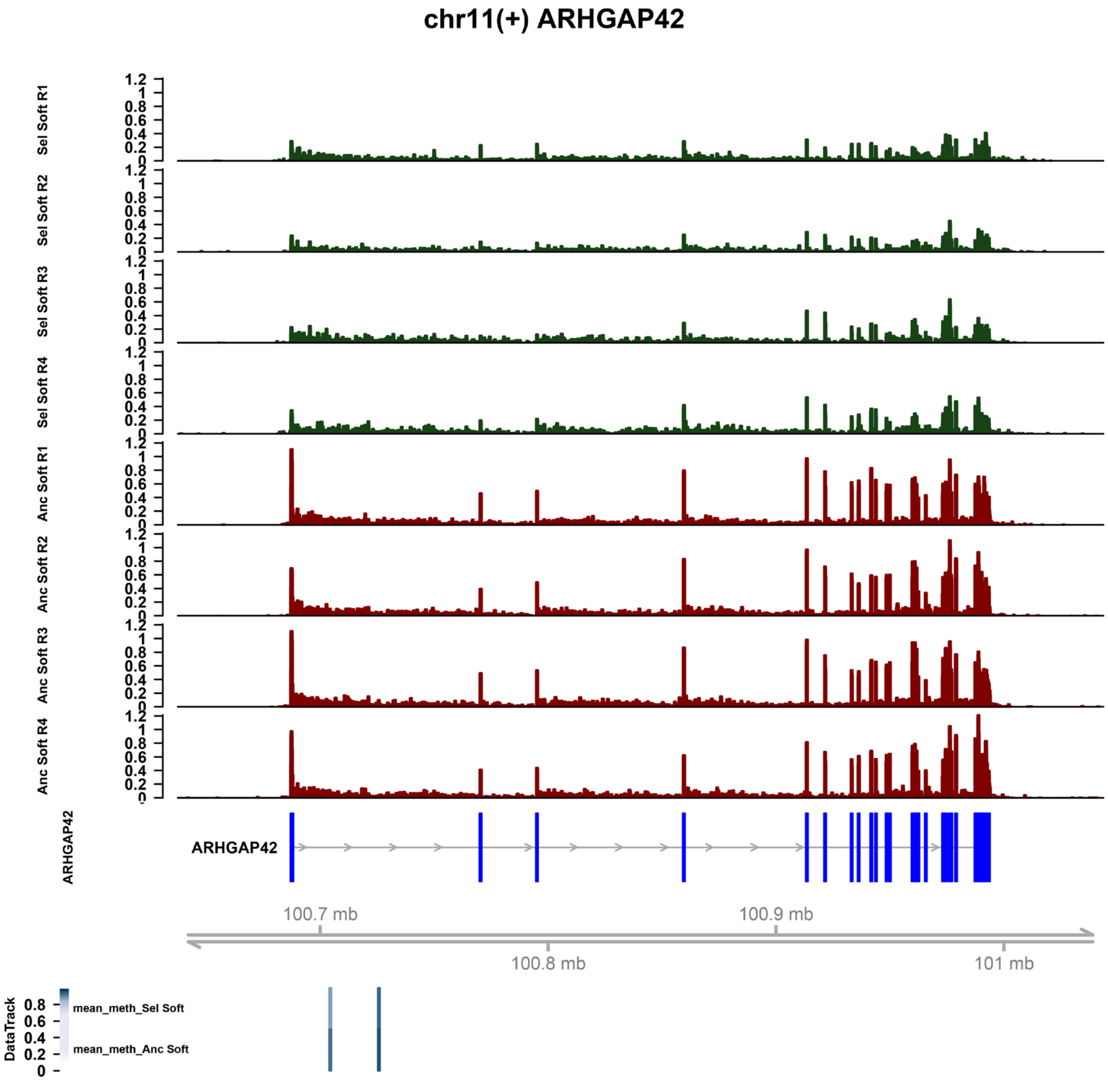

Fig. S4. RNA-seq and DNA methylation levels across the ARHGAP42 gene. The top eight tracks of the plot show the RNA-seq tracks, with four tracks for each group (soft-selected of soft ECM (Sel Soft; green) and ancestral on soft ECM (Anc Soft; red)). The last track shows the heatmap for mean methylation level for the significantly downregulated methylated regions in the ARHGAP42 gene (Region 1: chr11:100704298-10070460, p = 1.36 x 10^{-4}, FDR = 3.23 x 10^{-3}, logFC = -0.914; region 2: chr11:100704298-100704602, p = 1.97 x 10^{-4}, FDR = 4.33 x 10^{-3}, logFC = -1.105).
